# New Appliances and Things Medical

**Published:** 1897-09-18

**Authors:** 


### NEW APPLIANCES AND THINGS MEDICAL.

**[We shall be glad to receive, at our Office, 28 & 29, Southampton Street, Strand, London, W.C., from the manufacturers, specimens of all new preparations and appliances which may be brought out from time to time.]**

### VIFERA BURGUNDY.

(Robert Gemmell, 49, Virginia Street, Glasgow.)

We have made trial of this Burgundy, and find it a particularly pleasant wine, full-bodied and generous. For invalids and convalescents who require a stimulating and nourishing beverage we can thoroughly recommend this wine, partly on the ground of its comparative freedom from free acid, or saccharine bodies, and partly from the presence of those nitrogen-containing portions of the grape, which supplement the stimulating effects of the alcohol and give it the virtue of a more permanent tonic.

### THE CHRISTY ANATOMICAL SADDLE.

(A. Spalding and Bros., 54, Holborn Viaduct, E.C.)

The inventive are still busy over the vexed question of a perfect bicycle saddle. The saddle we have under consideration has many points in its favour if it cannot justly claim to have reached perfection. The position of the pads is good, and found to be very comfortable. The metal peak, which is entirely uncovered, is somewhat severe when quick riding is resorted to. In the case of the lady's saddle the peak is almost entirely absent, making the saddle so short and broad as to be inconvenient when riding down hill. We do not find that the division down the middle of the saddle is sufficiently wide to serve the purpose claimed for it. Further, we were not satisfied with the safety of the adjustment. However, Messrs. Spalding are a good way in the right direction to secure both a healthy and a convenient bicycle saddle, and a perusal of their small pamphlet clearly sets forth the dangers to be avoided when the choice of a saddle is made.

### N.A.P. (NATURAL WHOLE WHEAT) BREAD.

(Callard, Stewart, and Watt, Limited, 176, Piccadilly, W.)

The N.A.P. machine, which can be seen in working order at 176, Piccadilly, renders possible the manufacture of an agreeable and wholesome bread, containing all the proximate principles of the wheat grain in the natural proportions. The refined white bread of latter-day civilisation, although an elegant example of artistic manufacture, from a dietetic point of view will not stand a moment's comparison with the grain from which it has been laboriously manufactured. By the various processes of refining a large quantity of the phosphates and other valuable salts, albuminous and fatty ingredients, are removed, and the elegant remainder, from a dietetic standpoint, is little better than pure potato starch. The N.A.P. bread is in a certain sense a food in itself, of most valuable properties for nursery and invalid use; in some cases of diebetes it is highly appreciated.

